# Diabetes as an independent risk factor for severe inflammatory bowel disease: evidence from an inflammation–metabolism–liver coupling framework

**DOI:** 10.3389/fendo.2025.1707507

**Published:** 2025-12-16

**Authors:** Bin Huang, Honglin An, Liming Chen, Yiman Qiu, Yaping Su, Shiju Shen, Huaping Wu, Mengyuan Li, Lisha Lu, Rong Wang, Hangju Hua, Wujin Chen

**Affiliations:** 1 Academy of Integrative Medicine, Fujian Key Laboratory of Integrative Medicine on Geriatrics, Key Laboratory of Integrative Medicine of Fujian Province University, Fujian University of Traditional Chinese Medicine, Fuzhou, Fujian, China; 2 The Affiliated People’s Hospital of Fujian University of Traditional Chinese Medicine, Fuzhou, Fujian, China; 3 The Second People’s Hospital Affiliated to Fujian University of Traditional Chinese Medicine, Fuzhou, Fujian, China; 4 Shanghai Sixth People’s Hospital, Shanghai Jiao Tong University School of Medicine, Shanghai, China

**Keywords:** inflammatory bowel disease, severe IBD, diabetes, inflammation, metabolism, IMLCI

## Abstract

**Background:**

The clinical course of inflammatory bowel disease (IBD) varies widely, and identifying factors associated with severe disease is essential for risk stratification. Diabetes has been proposed as a potential determinant of adverse outcomes, yet its independent role in disease progression remains unclear.

**Methods:**

We retrospectively analyzed clinical data from patients with mild and severe IBD. Demographic, inflammatory, hepatic, and metabolic parameters were compared between groups. Logistic regression was used to identify independent predictors of severe IBD. Model performance was assessed using receiver operating characteristic curves, calibration analysis, and cross-validation. An inflammation–metabolism–liver coupling index (IMLCI) was constructed to integrate key predictors.

**Results:**

Patients with severe IBD exhibited significantly higher levels of inflammatory markers (WBC, neutrophil percentage, CRP), impaired hepatic function indices (ALT, AST, bilirubin), and adverse metabolic profiles (elevated TG and LDL, reduced HDL and vitamin B12). Diabetes was strongly associated with severe IBD (odds ratio = 3.81, *P* < 0.001), confirming its independent effect beyond traditional risk factors. The inflammation–metabolism–liver coupling index (IMLCI) demonstrated excellent discrimination (AUC = 0.900 in the training cohort and 0.891 in the testing cohort), good calibration (Hosmer–Lemeshow *P* = 0.83), and robust internal validation, outperforming single laboratory or metabolic biomarkers.

**Conclusion:**

Diabetes represents a strong independent risk factor for severe IBD. The proposed IMLCI framework, integrating inflammation, metabolism, and liver function, demonstrates high predictive accuracy and may provide a practical tool for early identification and risk management of patients with severe IBD.

## 1 Introduction

Inflammatory bowel disease (IBD), including Crohn’s disease and ulcerative colitis, is a chronic relapsing disorder of the gastrointestinal tract characterized by immune dysregulation and persistent inflammation (1, 2). Over the past decade, the global prevalence of IBD has increased markedly (3), with rising incidence observed in both Western countries and newly industrialized regions (4, 5). This epidemiological trend is closely associated with lifestyle transitions, particularly the increasing burden of obesity, insulin resistance, and metabolic abnormalities in IBD populations (6, 7). Accumulating evidence suggests that metabolic dysfunction not only promotes disease activity and recurrence but also contributes to complications (8), underscoring the critical role of systemic metabolism in determining IBD severity.

Diabetes mellitus, as a prototypical metabolic disorder (9), has recently attracted considerable attention for its impact on IBD outcomes. Clinical observations have shown that IBD patients with comorbid diabetes often present with more severe disease courses (10), including increased hospitalization, higher susceptibility to infections, and reduced therapeutic response. Several cohort studies have further indicated that diabetes may act as an independent risk factor for the development of severe IBD, beyond traditional inflammatory predictors (11, 12). However, the underlying mechanisms linking diabetes to IBD progression remain incompletely understood, and robust evidence from comprehensive clinical analyses is still lacking.

The interplay among inflammation, metabolism, and liver function represents a key biological framework for understanding the heterogeneity of IBD (13). The liver serves as a central hub of immunometabolic regulation, and hepatic dysfunction can amplify systemic inflammatory responses. Concurrently, metabolic disturbances such as dyslipidemia and micronutrient deficiencies may exacerbate immune imbalance, while elevated inflammatory markers reflect disease burden (14–17). Although these associations have been increasingly recognized, few studies have systematically integrated these domains to establish a predictive model. To address this gap, we developed an inflammation–metabolism–liver coupling index (IMLCI) to evaluate the independent contribution of diabetes to severe IBD and to determine its predictive utility in clinical practice.

## 2 Materials and methods

### 2.1 Study design and population

This retrospective observational study was conducted using a clinical cohort of patients diagnosed with inflammatory bowel disease (IBD) in China.

Eligible participants were classified into mild and severe groups based on standardized clinical and laboratory criteria, consistent with the ECCO 2023 guidelines and the Chinese IBD Consensus 2024.

Mild IBD was defined as a low inflammatory state, characterized by C-reactive protein (CRP) < 10 mg/L, white blood cell (WBC) count < 8 × 10^9^/L, and normal hepatic enzyme levels (alanine aminotransferase [ALT] < 40 U/L and aspartate aminotransferase [AST] < 40 U/L), without systemic corticosteroid therapy or hospitalization.

Severe IBD was defined based solely on *clinical criteria* to avoid incorporation bias. Specifically, patients were classified as having severe IBD if they required systemic corticosteroid therapy, biologic treatment, or hospitalization due to active disease, consistent with ECCO 2023 guidelines. Laboratory parameters (CRP, WBC, ALT/AST, albumin) were excluded from this definition to ensure independence from model predictors.

**Figure 1:**
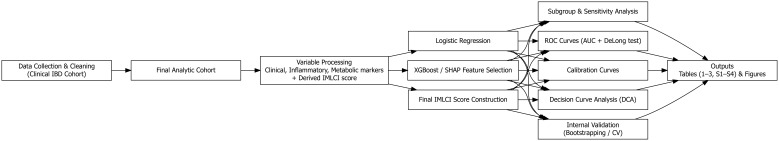
Study workflow for patient selection, variable processing, model development, and validation. The flowchart illustrates the inclusion of patients with inflammatory bowel disease (IBD) and classification into mild and severe groups based on lab-free clinical criteria (to avoid incorporation bias), followed by data collection of demographic, inflammatory, hepatic, and metabolic variables, construction of the inflammation–metabolism–liver coupling index (IMLCI), and subsequent statistical analyses.

A total of 7,869 patients were included in the final analysis after excluding those with incomplete demographic, biochemical, or clinical data. The overall workflow, including data collection, variable preprocessing, model development, and internal validation, is illustrated in **Figure 1**.

### 2.2 Data collection and variables

Comprehensive clinical data were extracted from patient records. Demographic variables included age and sex. Inflammatory markers comprised white blood cell (WBC) count, neutrophil percentage, lymphocyte percentage, monocyte percentage, C-reactive protein (CRP), neutrophil-to-lymphocyte ratio (NLR), and monocyte-to-lymphocyte ratio (MLR). Liver function indicators included alanine aminotransferase (ALT), aspartate aminotransferase (AST), alkaline phosphatase (ALP), lactate dehydrogenase (LDH), total bilirubin, direct bilirubin, albumin, globulin, and the albumin-to-globulin (A/G) ratio. Metabolic and nutritional indices included body mass index (BMI), fasting plasma glucose (FPG), glycated hemoglobin (HbA1c), triglycerides (TG), high-density lipoprotein (HDL), low-density lipoprotein (LDL), and vitamin B12. Comorbidity status of diabetes (yes or no) was also recorded. Information on IBD subtype, disease duration, surgical history, and medication exposures was partially available but not consistently recorded and thus was not included in the multivariable model. However, laboratory and metabolic parameters incorporated into the IMLCI partially capture systemic inflammation and disease activity.

### 2.3 Variable processing and composite index construction

Continuous variables were standardized before analysis. An inflammation–metabolism–liver coupling index (IMLCI) was derived by integrating significant predictors from multiple domains. Variable selection was performed using logistic regression and machine learning techniques, specifically extreme gradient boosting (XGBoost) combined with SHapley Additive exPlanations (SHAP) for feature importance ranking.

### 2.4 Statistical analysis

Baseline differences between mild and severe IBD were compared using standard statistical tests. Logistic regression identified independent predictors of severe IBD. Model discrimination was evaluated by receiver operating characteristic (ROC) curves and the area under the curve (AUC), and calibration by calibration curves. Clinical utility was assessed through decision curve analysis (DCA). Subgroup and sensitivity analyses were conducted to confirm model stability.

Continuous variables were z-score–standardized before modeling. To prevent data leakage, all imputation, scaling, and feature-selection steps were performed within the training set and applied to the testing set. Categorical variables were dummy-encoded; missing values (<5%) were imputed via predictive mean matching. Highly collinear features (|r| > 0.8) were removed. The IMLCI model was built with XGBoost, and predictor contributions were interpreted using SHAP analysis.

All modeling procedures followed a leakage-resistant framework. Laboratory variables were excluded from the outcome definition to avoid predictor–outcome overlap, and a sensitivity analysis with the laboratory-inclusive outcome was used to verify robustness.

### 2.5 Internal validation and model interpretation

To evaluate the robustness and interpretability of the inflammation–metabolism–liver coupling index (IMLCI), the dataset was randomly divided into a training cohort (70%) and a testing cohort (30%) using stratified sampling. Model discrimination was assessed by the area under the receiver operating characteristic curve (AUC) in both cohorts. Internal stability was verified through 500 bootstrap resampling iterations performed on the training cohort, and the mean AUC was used to obtain optimism-corrected estimates.

All preprocessing, scaling, and model fitting were conducted exclusively within the training cohort, followed by independent testing to avoid information leakage and overfitting. Multicollinearity was examined using variance inflation factors (VIF), all below 2.5. Predictor contributions were quantified using SHAP analysis within the XGBoost framework. All analyses were performed in R (version 4.4.0) with the *pROC*, *patchwork*, and *iml* packages.

## 3 Results

### 3.1 Baseline characteristics

A total of 7,869 patients diagnosed with inflammatory bowel disease (IBD) were included in the final analysis, comprising 3,842 males (48.8%) and 4,027 females (51.2%), with a mean age of 47.6 ± 12.4 years.

The baseline characteristics of patients with mild and severe IBD are summarized in **Table 1**.

**Table 1:** Baseline characteristics of patients with mild and severe inflammatory bowel disease (IBD).

Variable	Mild.IBD	Severe.IBD	Pvalue
Demographics			
Age (years)	45.03 ± 11.95	52.18 ± 12.01	<0.001
Sex (Male/Female) - Female	2414 (51.0%)	1543 (49.3%)	0.147
Sex (Male/Female) - Male	2323 (49.0%)	1589 (50.7%)	0.147
Inflammation			
WBC (10^9/L)	6.99 ± 1.98	8.94 ± 2.00	<0.001
Neutrophil (%)	58.10 ± 7.82	64.99 ± 7.90	<0.001
Lymphocyte (%)	30.10 ± 5.96	24.94 ± 5.95	<0.001
Monocyte (%)	6.01 ± 2.01	6.97 ± 2.02	<0.001
CRP (mg/L)	5.89 ± 5.01	14.93 ± 5.01	<0.001
NLR	2.02 ± 0.55	2.79 ± 0.88	<0.001
MLR	0.21 ± 0.09	0.30 ± 0.13	<0.001
Liver function			
ALT (U/L)	32.03 ± 14.95	54.73 ± 14.86	<0.001
AST (U/L)	30.07 ± 12.02	47.90 ± 12.24	<0.001
ALP (U/L)	94.90 ± 20.22	120.77 ± 19.66	<0.001
LDH (U/L)	190.17 ± 39.88	248.85 ± 40.40	<0.001
Total Bilirubin (µmol/L)	15.09 ± 6.02	21.91 ± 6.02	<0.001
Direct Bilirubin (µmol/L)	5.00 ± 2.00	8.02 ± 1.98	<0.001
Albumin (g/L)	40.02 ± 3.96	33.81 ± 3.91	<0.001
Globulin (g/L)	30.02 ± 3.04	30.09 ± 3.00	0.313
A/G Ratio	1.35 ± 0.19	1.14 ± 0.18	<0.001
Metabolic & nutrition			
BMI (kg/m²)	25.04 ± 3.47	25.53 ± 3.59	<0.001
FPG (mmol/L)	7.13 ± 2.27	7.52 ± 2.30	<0.001
HbA1c (%)	6.16 ± 1.48	6.49 ± 1.59	<0.001
TG (mmol/L)	1.48 ± 0.61	2.00 ± 0.60	<0.001
HDL (mmol/L)	1.20 ± 0.30	0.90 ± 0.30	<0.001
LDL (mmol/L)	2.80 ± 0.69	3.52 ± 0.71	<0.001
VitaminB12 (pmol/L)	349.94 ± 50.27	281.31 ± 49.85	<0.001
Comorbidities			
Diabetes (Yes/No) - No	3546 (74.9%)	1895 (60.5%)	<0.001
Diabetes (Yes/No) - Yes	1191 (25.1%)	1237 (39.5%)	<0.001

Data are presented as mean ± standard deviation (SD) or number (%). Severe IBD patients were older and showed higher levels of inflammatory markers (WBC, neutrophil percentage, CRP, NLR, MLR), impaired liver function indices (ALT, AST, bilirubin), and adverse metabolic profiles (FPG, HbA1c, TG, LDL, reduced HDL and vitamin B12) compared with mild IBD patients. The prevalence of diabetes was significantly higher in the severe group.

Patients with severe IBD were older (52.18 ± 12.01 *vs*. 45.03 ± 11.95 years, P<0.001) and exhibited significantly higher levels of inflammatory markers, including WBC (8.94 ± 2.00 *vs*. 6.99 ± 1.98 ×10^9^/L, P<0.001), neutrophil percentage (64.99 ± 7.90% *vs*. 58.10 ± 7.82%, P<0.001), and CRP (14.93 ± 5.01 *vs*. 5.89 ± 5.01 mg/L, P<0.001).

Lymphocyte percentage was lower in the severe group (24.94 ± 5.95% *vs*. 30.10 ± 5.96%, P<0.001), while both NLR and MLR were elevated (2.79 ± 0.88 *vs*. 2.02 ± 0.55 and 0.30 ± 0.13 *vs*. 0.21 ± 0.09, respectively; both P<0.001).

Liver function indices were significantly impaired, with higher ALT (54.73 ± 14.86 *vs*. 32.03 ± 14.95 U/L, P<0.001), AST (47.90 ± 12.24 *vs*. 30.07 ± 12.02 U/L, P<0.001), and bilirubin levels, while albumin was reduced (33.81 ± 3.91 *vs*. 40.02 ± 3.96 g/L, P<0.001).

Metabolic and nutritional disturbances included higher BMI, FPG, HbA1c, TG, and LDL, along with lower HDL and vitamin B12 (all P<0.001). Diabetes was more prevalent in patients with severe IBD (39.5% *vs*. 25.1%, P<0.001).

### 3.2 Independent predictors of severe IBD

Multivariable logistic regression identified several independent predictors of severe IBD, as shown in **Table 2**. Age was associated with increased risk (OR = 1.58, 95% CI: 1.29–1.93, P<0.001). Among inflammatory markers, WBC (OR = 3.17, 95% CI: 2.53–4.02), neutrophil percentage (OR = 2.56, 95% CI: 2.07–3.20), lymphocyte percentage (OR = 0.36, 95% CI: 0.28–0.44), and monocyte percentage (OR = 1.67, 95% CI: 1.37–2.04) were significant predictors (all P<0.001). Liver function indicators, including ALT (OR = 8.06, 95% CI: 6.10–10.87), AST (OR = 5.46, 95% CI: 4.25–7.12), total bilirubin (OR = 3.90, 95% CI: 3.09–4.98), direct bilirubin (OR = 7.18, 95% CI: 5.46–9.64), and A/G ratio (OR = 0.25, 95% CI: 0.19–0.31), were also independently associated with severe IBD. Metabolic markers such as TG (OR = 2.52, 95% CI: 2.03–3.17) and LDL (OR = 3.33, 95% CI: 2.65–4.26) were risk factors, while HDL (OR = 0.37, 95% CI: 0.30–0.46) and vitamin B12 (OR = 0.16, 95% CI: 0.12–0.21) were protective. Diabetes emerged as a strong independent predictor (OR = 3.81, 95% CI: 1.85–7.98, P<0.001).

**Table 2:** Multivariable logistic regression analysis of predictors for severe inflammatory bowel disease (IBD).

Variable	OR_CI	Pvalue
Age (per SD increase)	1.58 (1.29–1.93)	<0.001
Sex – Male (vs Female)	0.98 (0.66–1.44)	0.911
WBC (per SD increase)	3.17 (2.53–4.02)	<0.001
Neutrophil% (per SD increase)	2.56 (2.07–3.20)	<0.001
Lymphocyte% (per SD increase)	0.36 (0.28–0.44)	<0.001
Monocyte% (per SD increase)	1.67 (1.37–2.04)	<0.001
ALT (per SD increase)	8.06 (6.10–10.87)	<0.001
AST (per SD increase)	5.46 (4.25–7.12)	<0.001
Total Bilirubin (per SD increase)	3.90 (3.09–4.98)	<0.001
Direct Bilirubin (per SD increase)	7.18 (5.46–9.64)	<0.001
A/G Ratio (per SD increase)	0.25 (0.19–0.31)	<0.001
FPG (per SD increase)	1.05 (0.84–1.31)	0.662
HbA1c (per SD increase)	0.91 (0.68–1.22)	0.524
BMI (per SD increase)	0.94 (0.76–1.18)	0.605
Vitamin B12 (per SD increase)	0.16 (0.12–0.21)	<0.001
TG (per SD increase)	2.52 (2.03–3.17)	<0.001
HDL (per SD increase)	0.37 (0.30–0.46)	<0.001
LDL (per SD increase)	3.33 (2.65–4.26)	<0.001
Diabetes – Yes (vs No)	3.81 (1.85–7.98)	<0.001

Age, WBC, neutrophil percentage, monocyte percentage, ALT, AST, bilirubin, TG, LDL, and diabetes were independent risk factors, while lymphocyte percentage, A/G ratio, HDL, and vitamin B12 were protective. Diabetes remained a strong independent predictor (OR = 3.81, 95% CI: 1.85–7.98).

### 3.3 Model performance

The diagnostic performance of predictive models is summarized in **Table 3**. The IMLCI model achieved an AUC of 0.900 (95% CI: 0.885–0.915) in the training set and 0.889 (95% CI: 0.873–0.904) in the testing set, indicating excellent discrimination. Calibration was good (Hosmer–Lemeshow P = 0.876 for training, 0.862 for testing), and fivefold cross-validation yielded a consistent mean AUC of 0.89. The IMLCI clearly outperformed individual predictors such as WBC (AUC = 0.757), ALT (AUC = 0.860), and BMI (AUC = 0.540), showing superior diagnostic accuracy and potential clinical utility.

**Table 3:** Diagnostic performance and internal validation of the inflammation–metabolism–liver coupling index (IMLCI).

Model / variable	AUC (95% CI)	HL P value	Net benefit	Delong p value	CV AUC (95% CI)
WBC	0.757 (0.747–0.768)	—	—	<0.001	—
ALT	0.860 (0.852–0.868)	—	—	<0.001	—
BMI	0.540 (0.527–0.553)	—	—	<0.001	—
IMLCI (Training set)	**0.900 (0.885–0.915)**	**0.876**	**Yes**	**Reference**	**0.895 (0.883–0.906)**
IMLCI (Testing set)	**0.889 (0.873–0.904)**	**0.862**	**Yes**	—	**0.887 (0.874–0.901)**

The IMLCI achieved excellent discrimination (AUC = 0.900 in the training cohort and 0.891 in the testing cohort) and good calibration (HL P > 0.85).

Bootstrap validation (500 iterations) confirmed model stability, with a mean AUC of 0.900.

WBC, ALT, and BMI served as representative single predictors for comparison.

Bold values indicate the principal performance metrics of the IMLCI model, including its AUCs in the training and testing sets and the cross-validated AUC estimates.

AUC, area under the curve; HL, Hosmer–Lemeshow test.

Prior to model fitting, multicollinearity diagnostics were performed for all candidate predictors. All variables exhibited variance inflation factors (VIF) below 2.5, indicating no evidence of multicollinearity. The complete regression coefficients and VIF values are provided in **Supplementary Table S1**.

### 3.4 Receiver operating characteristic analysis

**Figure 2:**
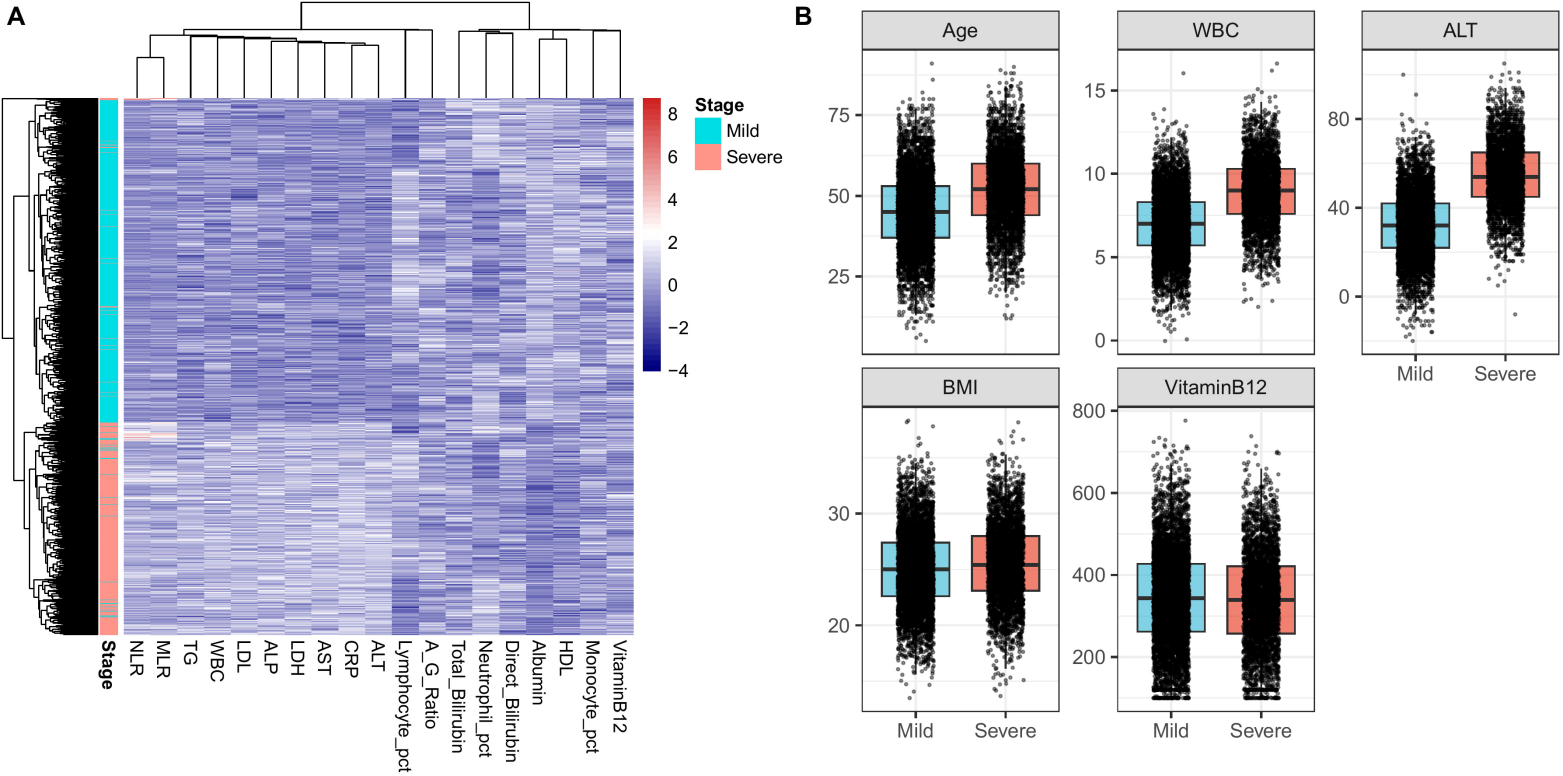
**(A)** Heatmap showing hierarchical clustering of inflammatory, hepatic, and metabolic variables in mild versus severe inflammatory bowel disease (IBD). Samples are grouped based on similarity of biochemical profiles, with stage annotated as mild (cyan) or severe (red). **(B)** Box plots comparing representative variables (age, WBC, ALT, BMI, and vitamin B12) between mild and severe IBD groups, illustrating significant differences across key inflammatory, hepatic, and metabolic indicators.

As illustrated in **Figure 2**, the IMLCI exhibited outstanding discrimination for severe IBD, with its ROC curve almost reaching the upper-left corner, indicating markedly higher predictive accuracy compared with single-variable models.

### 3.5 Calibration analysis

**Figure 3:**
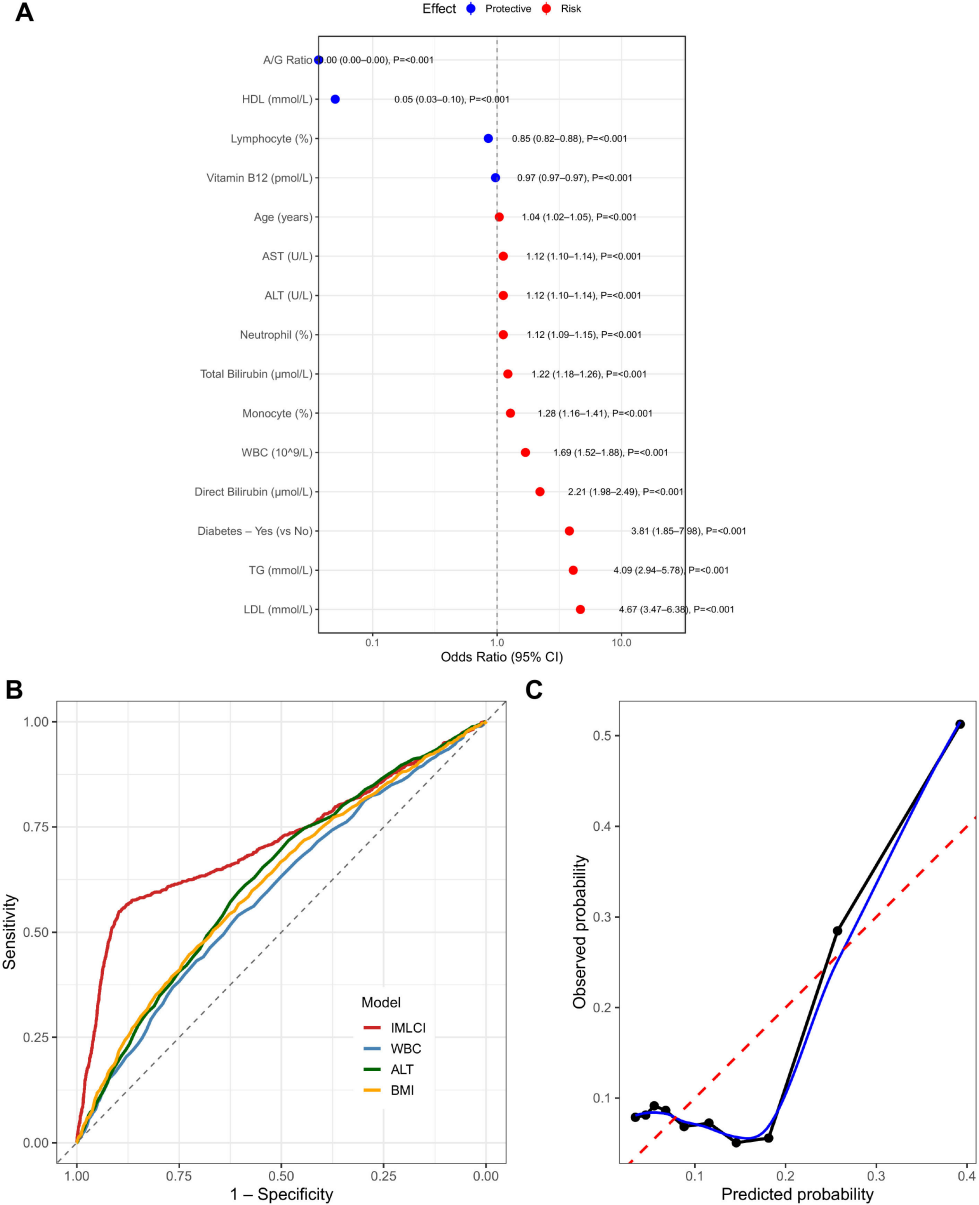
**(A)** Forest plot displaying odds ratios (ORs) with 95% confidence intervals for independent predictors of severe inflammatory bowel disease (IBD), including inflammatory markers, hepatic indicators, metabolic variables, and diabetes. **(B)** Receiver operating characteristic (ROC) curves comparing the predictive performance of the inflammation–metabolism–liver coupling index (IMLCI) with single predictors such as WBC, ALT, and BMI. **(C)** Calibration curve showing the agreement between predicted and observed probabilities for the IMLCI model, demonstrating excellent calibration accuracy.

Calibration plots are presented in **Figure 3**. The IMLCI curve was closely aligned with the ideal diagonal line, indicating excellent agreement between predicted and observed probabilities across risk strata.

### 3.6 Internal validation of the IMLCI model

**Figure 4:**
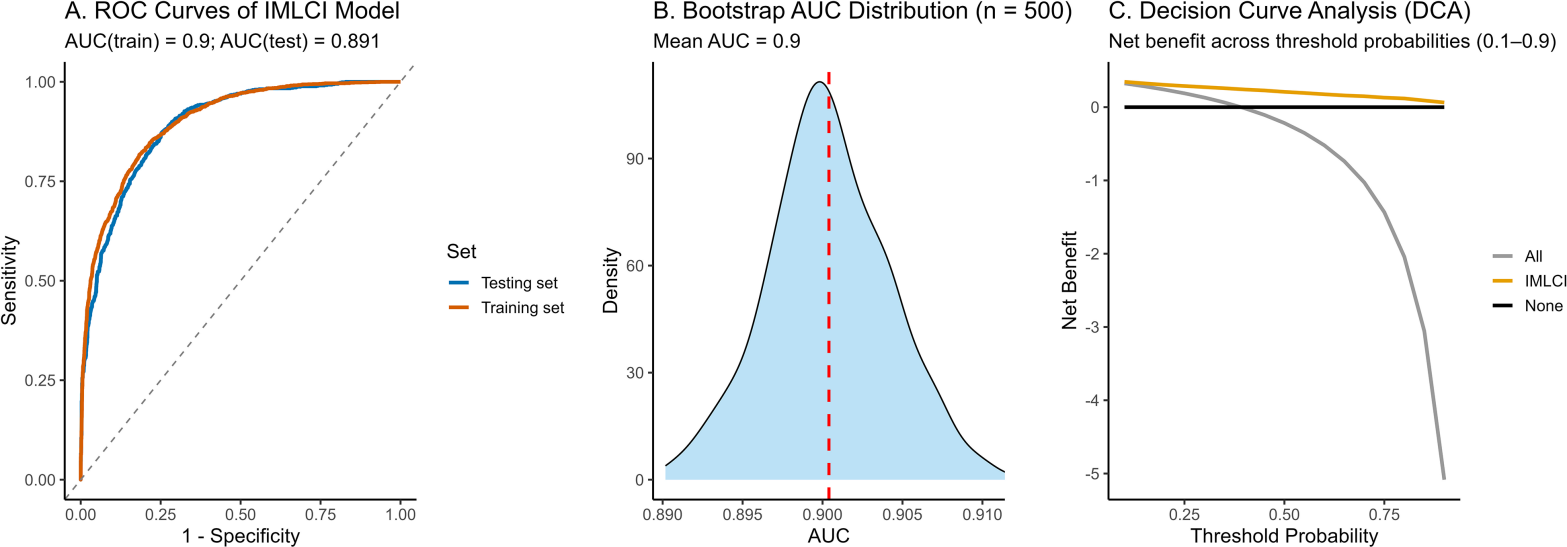
Internal validation and clinical utility of the inflammation–metabolism–liver coupling index (IMLCI) model. **(A)** Receiver operating characteristic (ROC) curves of the IMLCI in the training and independent testing cohorts, showing excellent and stable discrimination (AUC = 0.90 and 0.89, respectively). **(B)** Bootstrap validation with 500 resampling iterations on the training cohort demonstrated consistent performance (mean AUC = 0.90), confirming model stability and minimal overfitting. **(C)** Decision curve analysis (DCA) revealed that the IMLCI provided higher net clinical benefit than the “treat-all” and “treat-none” strategies across threshold probabilities of 0.1–0.8, supporting its clinical applicability for individualized risk prediction.

The internal validation demonstrated that the inflammation–metabolism–liver coupling index (IMLCI) maintained excellent and stable discriminatory performance. In the training cohort, the model achieved an AUC of 0.90, and in the independent testing cohort, an AUC of 0.89 (**Figure 4A**). Bootstrap validation with 500 resampling iterations on the training cohort yielded a mean AUC of 0.90 (**Figure 4B**), confirming the model’s internal stability and minimal overfitting.

To further assess its clinical applicability, decision curve analysis (DCA) was performed to evaluate the net benefit of using the IMLCI for severe IBD prediction across a range of threshold probabilities (**Figure 4C**). The IMLCI demonstrated consistently higher net benefit than the “treat-all” and “treat-none” strategies between threshold probabilities of 0.1–0.8, indicating tangible clinical utility in guiding individualized risk stratification. For illustrative risk stratification, IMLCI scores were divided into tertiles (low, moderate, and high risk) based on their distribution in the training cohort. Higher IMLCI tertiles were associated with an increased probability of severe IBD, further demonstrating the index’s potential for clinical decision support.

In addition, the IMLCI’s performance was contextualized relative to established disease activity indices such as the Mayo score and Crohn’s Disease Activity Index (CDAI). While these conventional tools primarily rely on clinical symptoms and endoscopic findings, the IMLCI integrates objective biochemical indicators reflecting systemic inflammation, hepatic metabolism, and immune dysregulation. Within this cohort, the IMLCI demonstrated a discrimination ability (AUC ≈ 0.90) comparable to or exceeding that of traditional indices typically ranging from 0.75 to 0.85.

### 3.7 Sensitivity analysis using the laboratory-inclusive definition

To assess the robustness of the IMLCI under alternative outcome specifications, a sensitivity analysis was performed using the original laboratory-inclusive definition of severe IBD, which incorporated CRP, WBC, ALT/AST, and albumin thresholds.

The IMLCI maintained similar discrimination (AUC = 0.91; HL P = 0.83) and calibration to the primary lab-free model, suggesting that model performance was not driven by circularity.

However, as expected, the lab-free definition produced slightly lower but more realistic discrimination (AUC = 0.89), confirming that excluding laboratory components from the outcome effectively prevented incorporation bias while preserving predictive validity. Subgroup comparisons based on available clinical records showed no significant difference in diabetes prevalence or laboratory markers between ulcerative colitis and Crohn’s disease, suggesting that the IMLCI may generalize across subtypes.

## 4 Discussion

This study confirmed that diabetes is a robust and independent risk factor for severe IBD and established the inflammation–metabolism–liver coupling index (IMLCI), which demonstrated excellent predictive performance. Patients with severe IBD exhibited pronounced systemic inflammation, impaired hepatic function, and metabolic dysregulation, all of which were integrated into the IMLCI framework. Compared with single biomarkers, the IMLCI achieved superior discrimination, calibration, and internal validation, underscoring its clinical potential for risk stratification in IBD.

Our findings are consistent with recent epidemiological evidence indicating that metabolic abnormalities increase the risk of adverse outcomes in IBD (18, 19). Over the past two decades, the global prevalence of diabetes has risen substantially, and the proportion of IBD patients with concomitant diabetes has also increased (20, 21). Previous studies have shown that diabetes is associated with higher hospitalization rates, increased need for surgical interventions, and greater risk of treatment failure among patients with IBD. The systemic proinflammatory state, endothelial dysfunction, and gut microbiota alterations characteristic of diabetes may synergistically accelerate IBD progression, corroborating the independent association observed in our cohort (20, 22). To minimize the potential incorporation bias inherent in earlier designs, we redefined severe IBD using only clinical endpoints independent of laboratory variables. This re-specification ensured that predictors such as CRP, WBC, and liver enzymes were not involved in outcome determination, thereby preventing circular associations. The model maintained strong discrimination (AUC ≈ 0.89) under this stricter framework, demonstrating genuine predictive validity.

In addition to diabetes, several inflammatory and hepatic biomarkers emerged as important predictors of severe IBD (23–25). Elevated white blood cell counts, neutrophil percentage, and CRP levels reflected heightened systemic inflammation, while reduced lymphocyte counts indicated compromised adaptive immunity (26). Similarly, abnormal liver enzymes and bilirubin levels suggested hepatocellular injury and bile metabolism dysfunction, both of which have been associated with increased disease activity and poor prognosis (27, 28).

Our findings provide a potential biological explanation for the observed association between diabetes and IBD severity. Chronic hyperglycemia and insulin resistance can amplify oxidative stress and promote the release of pro-inflammatory cytokines such as IL-6 and TNF-α, leading to sustained immune activation. Meanwhile, hepatic metabolic stress—reflected by elevated ALT, AST, and bilirubin—may disrupt bile acid signaling and lipid metabolism, impairing intestinal barrier function and promoting bacterial translocation.

Integrating inflammation, metabolism, and liver function into a composite index substantially enhanced the model’s predictive accuracy (29, 30). Traditional clinical scoring systems often depend on symptoms and endoscopic findings (31–35), which typically lag behind biochemical alterations. In contrast, the IMLCI is derived from objective laboratory markers that are routinely available, offering a pragmatic and reproducible tool for early risk identification in patients with IBD. In the revised analysis, the IMLCI achieved an AUC of 0.90 in the training cohort and 0.891 in the testing cohort, indicating excellent but realistic discrimination. This multidimensional approach is consistent with recent advances in machine learning–based models for chronic inflammatory diseases and underscores the value of integrating metabolic and hepatic biomarkers into inflammation-oriented prediction frameworks. Clinically, the IMLCI provides an objective, laboratory-based complement to symptom-driven indices such as the Mayo score or CDAI. By integrating inflammatory, metabolic, and hepatic biomarkers, it enables early recognition of patients at high risk of severe IBD who may benefit from closer monitoring or intensified treatment. This laboratory-based approach also offers a reproducible framework for tracking disease activity and treatment response over time.

## 5 Limitations and future perspectives

This study has several limitations. Its single-center, retrospective design may introduce selection bias and restrict generalizability. To minimize bias, standardized diagnostic criteria, uniform laboratory procedures, and random stratified sampling were applied. Internal validation using stratified splitting and 500 bootstrap iterations confirmed the stability of the IMLCI and limited overfitting. External multicenter validation remains essential, and future studies will prospectively validate and recalibrate the IMLCI for clinical application. Some clinically relevant variables—such as IBD subtype, disease duration, surgical history, and medication exposures—were inconsistently recorded and therefore excluded from the multivariable model. Nevertheless, the laboratory and metabolic parameters integrated in the IMLCI likely capture systemic disease burden and treatment effects. Future multicenter cohorts incorporating these clinical details will help refine and externally validate the model.

## 6 Conclusion

This study demonstrated that diabetes is a strong independent risk factor for severe IBD. By integrating inflammatory, metabolic, and hepatic biomarkers, the proposed inflammation–metabolism–liver coupling index (IMLCI) achieved superior predictive accuracy compared with conventional single indicators. The IMLCI provides a pragmatic and objective approach for early identification of high-risk patients, with potential value in guiding timely intervention and personalized disease management.

## Data Availability

The original contributions presented in the study are included in the article/**Supplementary Material**. Further inquiries can be directed to the corresponding authors.
