# Supplementary material for: Diabetes as an independent risk factor for severe inflammatory bowel disease: evidence from an inflammation–metabolism–liver coupling framework
Source: Front Endocrinol (Lausanne). 2025 Dec 16;16:1707507. doi: 10.3389/fendo.2025.1707507 (PMC12747988; doi:10.3389/fendo.2025.1707507)
Supplement: Supplementary file 1 [file DataSheet1.docx]

| Variable | OR_CI | Pvalue | VIF |
| --- | --- | --- | --- |
| Age (per SD increase) | 1.58 (1.29–1.93) | <0.001 | 1.03 |
| Sex – Male (vs Female) | 0.98 (0.66–1.44) | 0.911 | NA |
| WBC (per SD increase) | 3.17 (2.53–4.02) | <0.001 | 1.15 |
| Neutrophil% (per SD increase) | 2.56 (2.07–3.20) | <0.001 | 1.08 |
| Lymphocyte% (per SD increase) | 0.36 (0.28–0.44) | <0.001 | 1.11 |
| Monocyte% (per SD increase) | 1.67 (1.37–2.04) | <0.001 | 1.05 |
| ALT (per SD increase) | 8.06 (6.10–10.87) | <0.001 | 1.3 |
| AST (per SD increase) | 5.46 (4.25–7.12) | <0.001 | 1.16 |
| Total Bilirubin (per SD increase) | 3.90 (3.09–4.98) | <0.001 | 1.13 |
| Direct Bilirubin (per SD increase) | 7.18 (5.46–9.64) | <0.001 | 1.22 |
| A/G Ratio (per SD increase) | 0.25 (0.19–0.31) | <0.001 | 1.19 |
| FPG (per SD increase) | 1.05 (0.84–1.31) | 0.662 | 1.33 |
| HbA1c (per SD increase) | 0.91 (0.68–1.22) | 0.524 | 2.4 |
| BMI (per SD increase) | 0.94 (0.76–1.18) | 0.605 | 1.37 |
| Vitamin B12 (per SD increase) | 0.16 (0.12–0.21) | <0.001 | 1.21 |
| TG (per SD increase) | 2.52 (2.03–3.17) | <0.001 | 1.09 |
| HDL (per SD increase) | 0.37 (0.30–0.46) | <0.001 | 1.05 |
| LDL (per SD increase) | 3.33 (2.65–4.26) | <0.001 | 1.16 |
| Diabetes – Yes (vs No) | 3.81 (1.85–7.98) | <0.001 | NA |

**Supplementary Table S1. Multivariable logistic regression coefficients and multicollinearity diagnostics of the inflammation–metabolism–liver coupling index (IMLCI) model.**
This table presents the complete multivariable logistic regression results for predictors of severe inflammatory bowel disease (IBD). Odds ratios (OR) are expressed per standard deviation (SD) increase in each continuous variable, with corresponding 95% confidence intervals (CI) and P values. Variance inflation factors (VIF) were calculated to assess multicollinearity. All predictors demonstrated VIF < 2.5, indicating no evidence of collinearity.

| IMLCI_risk_group | N | Severe_IBD_n | Severe_IBD_pct | Mean_IMLCI | SD_IMLCI | Range_IMLCI |
| --- | --- | --- | --- | --- | --- | --- |
| Low | 2576 | 106 | 4.1 | -1.104 | 0.463 | -3.68 – -0.50 |
| Moderate | 2627 | 878 | 33.4 | -0.044 | 0.262 | -0.50 – 0.43 |
| High | 2666 | 2148 | 80.6 | 1.11 | 0.514 | 0.43 – 4.13 |

**Supplementary Table S2.**
Distribution of patients across tertile-based risk categories derived from the inflammation–metabolism–liver coupling index (IMLCI).
The cohort was stratified into low-, moderate-, and high-risk groups according to tertile cut-points of IMLCI scores calculated in the training dataset.
Patients in the high-risk tertile exhibited a markedly higher prevalence of severe inflammatory bowel disease (80.6%), compared with 33.4% in the moderate-risk and 4.1% in the low-risk group.
Mean IMLCI scores increased progressively across tertiles (–1.104, –0.044, and 1.110), confirming the index’s strong gradient association with disease severity.
These findings support the IMLCI as a robust and clinically interpretable tool for individualized risk stratification.

Code:

suppressPackageStartupMessages({

library(dplyr)

library(openxlsx)

})

data <- read.csv("D:/Frontiers In Endocrinology/清洗好的数据.csv")

data$Severe_IBD <- ifelse(data$IBD_stage == "Severe", 1, 0)

data$IMLCI_score <- with(data,

+0.45 * scale(WBC) + 0.35 * scale(Neutrophil_pct) + 0.25 * scale(Monocyte_pct)

- 0.30 * scale(Lymphocyte_pct)

+ 0.40 * scale(ALT) + 0.40 * scale(AST)

+ 0.35 * scale(Total_Bilirubin) + 0.35 * scale(Direct_Bilirubin)

+ 0.30 * scale(TG) + 0.30 * scale(LDL) - 0.30 * scale(HDL)

- 0.35 * scale(A_G_Ratio) - 0.25 * scale(VitaminB12)

+ 0.25 * scale(Age) + 0.5 * ifelse(Diabetes == "Yes", 1, 0)

)

data$IMLCI_score <- as.numeric(scale(data$IMLCI_score))

set.seed(2025)

train_idx <- sample(seq_len(nrow(data)), size = 0.7 * nrow(data))

train <- data[train_idx, ]

cut_points <- quantile(train$IMLCI_score, probs = c(1/3, 2/3), na.rm = TRUE)

data <- data %>%

mutate(IMLCI_risk_group = case_when(

IMLCI_score <= cut_points[1] ~ "Low",

IMLCI_score <= cut_points[2] ~ "Moderate",

TRUE ~ "High"

))

risk_summary <- data %>%

group_by(IMLCI_risk_group) %>%

summarise(

N = n(),

Severe_IBD_n = sum(Severe_IBD, na.rm = TRUE),

Severe_IBD_pct = round(100 * mean(Severe_IBD, na.rm = TRUE), 1),

Mean_IMLCI = round(mean(IMLCI_score, na.rm = TRUE), 3),

SD_IMLCI = round(sd(IMLCI_score, na.rm = TRUE), 3),

Range_IMLCI = sprintf("%.2f – %.2f",

min(IMLCI_score, na.rm = TRUE),

max(IMLCI_score, na.rm = TRUE))

) %>%

arrange(factor(IMLCI_risk_group, levels = c("Low", "Moderate", "High")))

out_path <- "D:/Frontiers In Endocrinology/Supplementary_Table_S2_IMLCI_RiskStratification.xlsx"

write.xlsx(risk_summary, out_path, rowNames = FALSE)

print(risk_summary)

cat("\nSupplementary Table S2 successfully saved to:\n", out_path, "\n")
